# Effect of esomeprazole on the pharmacokinetics of carbamazepine

**DOI:** 10.4103/0253-7613.75675

**Published:** 2011-02

**Authors:** Bikash Medhi, Pawan K. Singh, Ajay Prakash, Pramod Avti

**Affiliations:** Department of Pharmacology, Postgraduate Institute of Medical Education and Research, Chandigarh, India

**Keywords:** Carbamazepine, esomeprazole, pharmacokinetics

## Abstract

**Objective:**

Present study was carried out to evaluate effect of esomeprazole on the pharmacokinetics of carbamazepine in rabbits.

**Materials and Methods:**

Study was conducted at Department of Pharmacology, Postgraduate Institute of Medical Education and Research from March to October 2007. In a parallel design study, carbamazepine 40 mg/kg/day was given orally for 14 days. On day 15, blood samples were taken at various time intervals between 0 and 24 hours. In esomeprazole group, carbamazepine was administered for 14 days as above. On day 8, esomeprazole 2.8 mg/kg/day along with carbamazepine 40 mg/kg/day was administered till 14 days and blood samples were drawn on 15^th^ day. Plasma levels of carbamazepine were assayed by high-performance liquid chromatography and pharmacokinetic parameters were calculated.

**Results:**

In all groups there was a decrease in the AUC_0-24_ when carbamazepine was coadministered with esomeprazole. The decrease in AUC_0-24_ (22.78 ± 4.71 to 10.46 ± 2.29), C_max_ (2.76 ± 0.77 to 1.412±1.08), T_max_ (2.83 ± 0.17 to 3 ± 0.40) was statistically significant (*P* < 0.05) when esomeprazole was given along with carbamazepine. Additionally, absorption and elimination constant were also altered significantly.

**Conclusions:**

These results suggest that concomitant use of esomeprazole alters the pharmacokinetics of carbamazepine. Confirmation of these results in human studies will warrant changes in carbamazepine dose or frequency when esomeprazole is coadministered.

## Introduction

Carbamazepine is one of the most commonly prescribed antiepileptic drugs. It is used on a long-term basis for control of generalized tonic-clonic seizures. Carbamazepine is metabolized by CYP3A4 and can also induce this cytochrome enzyme.[1] In rabbits, CYP3A6 is the cytochrome responsible for carbamazepine metabolism and corresponds to CYP3A4 activity in human hepatocytes.[2]

Proton pump inhibitors (PPI) are one of the most commonly prescribed class of medications and are considered a major advance in the treatment of acid-peptic diseases including gastroesophageal reflux disorder, peptic ulcer disease, and nonsteroidal anti-inflammatory drug-induced gastropathy. These drugs have minimal side effects, few significant drug interactions and are considered safe for long-term treatment. The PPIs omeprazole, lansoprazole, rabeprazole, and the recently approved esomeprazole appear to have similar efficacy.[3] Esomeprazole, the (S)-isomer of omeprazole, is the first PPIs developed as a single isomer for the treatment of acid-peptic diseases. Because of its extensive use, the documentation of the probability for drug interactions is of great importance. Esomeprazole is extensively metabolized by CYP3A4 and CYP2C19 isoenzyme in the liver[4] Thus, it is apparent that the carbamazepine share same metabolic pathways with esomeprazole. Depending on ethnic variations, metabolism of a drug by a particular cytochrome may predominate. So, the potential of drug interactions between these two groups of drugs exist. Moreover, the high incidence of gastrointestinal disorders and epilepsy may imply the existence of one in the presence of the other. Epilepsy is a chronic disease requiring long-term treatment. An episode of gastrointestinal problem in an epileptic patient on carbamazepine will necessitate the coadministration of these antiepileptics with esomeprazole. The drug interactions of carbamazepine and esomeprazole in these situations will be important to the practicing physician. So far, no significant interactions have been reported. So, the purpose of this study was to evaluate the effect of esomeprazole on the pharmacokinetics of carbamazepine.

## Materials and Methods

The study was conducted at Department of Pharmacology, Postgraduate Institute of Medical Education and Research, Chandigarh from March to October 2007 and approved by the Institute Animal Ethics Committee (IAEC). Randomly selected healthy male white New Zealand rabbits weighing between 1.5 and 2.5 kg were included in the study. The rabbits were kept under standard animal house conditions of 12 hours day-night cycle at a temperature of 25±2°C and humidity of 60±2%. The animals were allowed to take water *ad libitum* and free access to standard diet. The blood samples were withdrawn after application of topical lignocaine 4% anesthesia to minimize pain.

Carbamazepine (IPCA Lab, Mumbai, India) and esomeprazole (Lupin Pharmaceuticals, Mumbai, India) were in the bulk powder form and dissolved in appropriate solvents just before administration.

A parallel study design with six rabbits in each group was used. In control group, six rabbits were administered 40 mg/kg/day carbamazepine per oral at 0800 hours for 14 consecutive days using an orogastric tube. On day 15, blood samples (1 ml) were collected before administration of next dose of carbamazepine at 0 hr and then at 0.5, 1, 2, 3, 4, 5, 6, 9, 12, and 24 hours after drug administration. In esomeprazole group, in addition to the same dose of carbamazepine, from 8^th^ day onward oral esomeprazole 0.26 mg/kg/day was administered till 14 days. Blood samples were drawn at similar time intervals as mentioned above. All blood samples were drawn from the marginal ear vein after topical anesthesia with 4% lignocaine solution. Each sample was collected in labeled, heparinized test tubes and centrifuged at 3000 rpm for 10 min. Plasma was separated by centrifugation and stored at -20°C until assayed for carbamazepine by high-performance liquid chromatography (HPLC) method.

### 

#### Extraction procedure:

To 0.2 ml plasma sample/standard sample 0.2 ml of 1.0 M of sodium acetate buffer (pH 5.5) and 3.0 ml of chloroform were added. The tubes were shaken for 1 min and then centrifuged at 3000 rpm for 10 min. Following this, 2.8 ml of chloroform layer was transferred in another test tube and the chloroform was evaporated at 50°C on a water bath. The residue was reconstituted in 0.2 ml of mobile phase to be used for HPLC assay. One hundred microliters of this reconstituted solution was injected to HPLC system for assay.

#### HPLC conditions:

The mobile phase was acetonitrile: methanol: 4 mM potassium phosphate buffer (pH 6.0) in ratio of 20:40:40(V/V/V) delivered at a flow rate of 1.0 ml/min at ambient temperature. Absorbance was measured using a UV detector at 215 nm at a sensitivity of 0.02 AUFS. The sensitivity of the assay was 0.1 *μ*g/ml with recovery 98% or more. The standards used for carbamazepine ranged from 0.25 to 32 *μ*g/ml.[5]

Peak plasma concentration (C_max_) and time to reach the peak plasma concentration (T_max_) were calculated from the actual plasma level data. Rate constant for plasma drug elimination i.e. K_el_ was calculated by regression analysis of the monoexponential declining line of the log of plasma drug concentration versus time curve, while elimination half life (t_½el_) was obtained from the formula, t_½el_ =0.693/K_el_. Absorption rate constant K_a_ was calculated by the method of residuals. The absorption half life (t_½a_) was calculated from the formula t_½a_ = 0.693/K_a_ .Area under the plasma drug concentration versus time curve (AUC_0-t_) was calculated by trapezoidal rule. Extension of the AUC data to infinity (AUC_t-∞_) was done by dividing the last observed concentration in plasma by the elimination rate constant (K_el_). The AUC_0-∞_ was calculated from the sum of AUC_0-t_ and AUC_t-∞_. Statistical analysis was done using the Student’s paired t-test and *P* < 0.05 was considered statistically significant.

## Results

**Figure 1:**
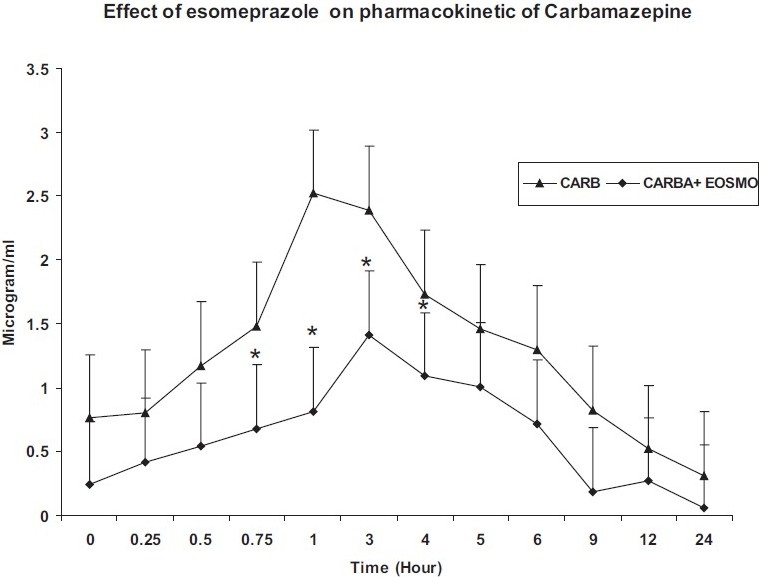
Plasma levels (mean±SEM) of carbamazepine alone and in combination with esomeprazole at different time intervals in rabbit.

The mean plasma levels were evaluated for the carbamazepine alone and in combination with esomeprazole. The plasma level of carbamazepine was significantly decreased in esomeprazole treated group; AUC_0-24_ was (22.78±4.71 vs 10.46 ± 12.29) (*P* < 0.05), T_max_ (2.83 ± 0.17 vs 3±0.40 hr), K_el_ (0.1216 ± 0.06 vs 0.0966 ± 0.04), C_max_ (2.76± 0.77 to 1.412±1.08), and t_½a_ (2.36±0.66 vs 1.82±0.25) (*P* < 0.05) [Table 1]. However, K_a_ (0.2936 ± 0.09 vs 0.3939 ± 0.03) was increased significantly (*P* < 0.05) in the esomeprazole and carbamazepine treated group compared with carbamazepine alone group [Figure 1].

**Table 1:** Pharmacokinetic parameters following oral administration of carbamazepine alone and in combination with esomeprazole in rabbits

*Pharmacokinetic parameters*	*Carbamazepine pharmacokinetics*	
	*Carbamazepine alone*	*With esomeprazole*
C_max_ (μg/ml)	2.76 ± 0.77	1.412 ± 1.08*
T_max_ (hrs)	2.83 ± 0.17	3.0 ± 0.40
t_½ a_ (hrs)	2.36 ± 0.66	1.82 ± 0.25*
Ka	0.2936 ± 0.09	0.3939 ± 0.03*
Kel	0.1216 ± 0.06	0.0966 ± 0.04
t_½ el_ (hrs)	5.70 ± 1.14	7.181 ± 0.40*
AUC_0-24_ (μg/ml.h)	22.78 ± 4.71	10.46 ± 12.29*

Values are mean ± SEM, (n=6); *P* < 0.05

* *P* < 0.05

## Discussion

This study showed the concomitant use of esomeprazole along with carbamazepine significantly decrease plasma levels of carbamazepine. This indicates an enhancement of carbamazepine metabolism leading to lower plasma level of carbamazepine. There was a significant decrease of C_max_ of carbamazepine by 51.16% in the esomeprazole group as compared with control (carbamazepine alone group). The absorption also increased in the esomeprazole group. Such a variation would lead to subtherapeutic concentrations and a consequent lack of therapeutic efficacy of carbamazepine. Previous studies suggested an interaction between esomeprazole and carbamazepine, where carbamazepine levels were increased in the presence of esomeprazole and omeprazole.[6] But, the present study contradicts the previous findings. The AUC_0-24_ of carbamazepine decreased by about two-fold to that of control values when esomeprazole was coadministered. Esomeprazole is metabolized by CYP2C19 and CYP3A4 and carbamazepine is metabolized by CYP3A4 which can lead to competitive enhancement of carbamazepine metabolism.[3] But, the present results showed statistically significant difference and probably reflects the effect of auto-induction by carbamazepine;[1] as it induces CYP3A4 and increases its own metabolism. Whether the ultimate effect on plasma levels is important enough to warrant dose adjustment of carbamazepine, actually needs clinical evaluation.

The interaction between carbamazepine and esomeprazole is not reported till now. The basis for studying the interaction between carbamazepine and esomeprazole was the common metabolic pathway involving the isoenzyme CYP3A4. In rabbits, it has been seen that the isoenzymes CYP3A6 corresponds to CYP3A4 activity in human hepatocytes.[2] Hence, drugs like carbamazepine that are metabolized by CYP3A4 in humans will be biotransformed by CYP3A6 in rabbits. Any drug interaction occurring due to an effect on this particular cytochrome i.e., CYP3A6 will correlate to an interaction at CYP3A4 levels in humans.

This is the first study to report the interaction between the antiepileptic carbamazepine with esomeprazole. Owing to ethical constraints, a minimum number of animals were used. However, the importance of these findings should not be ignored. Carbamazepine is one of the most commonly used antiepileptic drugs and also indicated for trigeminal neuralgia and alcohol dependence. Thus, there is a significant percentage of population who require this drug in their life time. Esomeprazole is most commonly prescribed in clinical practice because of its superior efficacy in peptic ulcer patients.[3] Genetic polymorphisms account for variations in metabolism of antiepileptic drugs in different ethnic populations.[6–9] Therefore, predominant metabolism may occur by a particular subtype of cytochrome. In addition, the search for identification of the complete set of cytochromes responsible for esomeprazole metabolism needs further research.

In conclusion, present study showed that esomeprazole can alter the pharmacokinetics of carbamazepine to statistically significant levels. However, further confirmation of these findings is required in human studies using larger sample size before these results are applied in patient care.
